# A Metagenomic Investigation of Potential Health Risks and Element Cycling Functions of Bacteria and Viruses in Wastewater Treatment Plants

**DOI:** 10.3390/v16040535

**Published:** 2024-03-29

**Authors:** Haozhe Zhao, Mingfei Yang, Xiang Fan, Qian Gui, Hao Yi, Yigang Tong, Wei Xiao

**Affiliations:** 1 Yunnan Institute of Microbiology, School of Life Sciences, Yunnan University, Kunming 650091, China; zhaohaozhe@stu.ynu.edu.cn (H.Z.); yangmingfei@stu.ynu.edu.cn (M.Y.); ljiu184@gmail.com (X.F.); guiqian@stu.ynu.edu.cn (Q.G.); yihao@stu.ynu.edu.cn (H.Y.); 2 College of Life Science and Technology, Beijing University of Chemical Technology, Beijing 100029, China

**Keywords:** wastewater treatment plants, virus, prokaryote, element cycling, health risks

## Abstract

The concentration of viruses in sewage sludge is significantly higher (10–1000-fold) than that found in natural environments, posing a potential risk for human and animal health. However, the composition of these viruses and their role in the transfer of pathogenic factors, as well as their role in the carbon, nitrogen, and phosphorus cycles remain poorly understood. In this study, we employed a shotgun metagenomic approach to investigate the pathogenic bacteria and viral composition and function in two wastewater treatment plants located on a campus. Our analysis revealed the presence of 1334 amplicon sequence variants (ASVs) across six sludge samples, with 242 ASVs (41.22% of total reads) identified as pathogenic bacteria. *Arcobacter* was found to be the most dominant pathogen accounting for 6.79% of total reads. The virome analysis identified 613 viral genera with *Aorunvirus* being the most abundant genus at 41.85%. Approximately 0.66% of these viruses were associated with human and animal diseases. More than 60% of the virome consisted of lytic phages. Host prediction analysis revealed that the phages primarily infected *Lactobacillus* (37.11%), *Streptococcus* (21.11%), and *Staphylococcus* (7.11%). Furthermore, our investigation revealed an abundance of auxiliary metabolic genes (AMGs) involved in carbon, nitrogen, and phosphorus cycling within the virome. We also detected a total of 113 antibiotic resistance genes (ARGs), covering major classes of antibiotics across all samples analyzed. Additionally, our findings indicated the presence of virulence factors including the *clpP* gene accounting for approximately 4.78%, along with toxin genes such as the *RecT* gene representing approximately 73.48% of all detected virulence factors and toxin genes among all samples analyzed. This study expands our understanding regarding both pathogenic bacteria and viruses present within sewage sludge while providing valuable insights into their ecological functions.

## 1. Introduction

Viruses are the most abundant entities on earth, and the number of viruses is estimated to be as high as 10^31^ [1,2]. The rapid development of metaviromic technology in the past decade has promoted the study of viruses in a variety of environments, including marine, freshwater, and intestinal tract environments, revealing many new viral species and a large amount of functional information. Among these viruses, studies on the marine virome have revealed that viruses play an indispensable role in the regulation of host communities, biogeochemical cycles, global climate change, and horizontal gene transfer [3]. Sewage sludge harbours enormous quantities of viruses, with 10–1000-fold higher concentrations than in natural environments, and some of these viruses can cause severe human and animal diseases. Furthermore, a limited number of studies to date have investigated sewage treatment plants. Despite the increasing application of high-throughput sequencing techniques for viral metagenomics, their use in identifying viruses in sewage has not been well explored [4,5,6]. However, the few existing studies have demonstrated that viruses are key factors in nutrient removal and that sewage sludge, as a reservoir of viruses, may pose threats to human health. The sewage virosphere has been monitored worldwide using molecular techniques, such as PCR and quantitative PCR [5,7]. These methods can only provide information about the presence and abundance of known and characterized viruses because there is no universal marker for viruses, such as the 16S rRNA gene for bacteria [4,5].

Antibiotic resistance is a growing global public health challenge and is expected to cause a difference in the burden on low- and middle-income countries in the next few decades [8]. Wastewater and wastewater treatment systems are important reservoirs of antibiotic-resistant bacterial populations, which contribute to regular outbreaks of drug-resistant infections [9]. Metaviromic technology can not only reveal new viruses but also provides the possibility for understanding the ecological functions of viruses. Metaviromic methods can be used to effectively detect and monitor the transfer of harmful genes, such as ARGs, virulence factors, and toxin genes. Previous studies have focused more on the function of bacteria in wastewater treatment systems and have ignored the regulatory role of viruses in the function of bacteria. In fact, the auxiliary metabolic genes contained in viruses are important regulators of host metabolism and regulate the metabolic function of the host during interaction with the host, thus affecting biogeochemical cycle processes [10].

Considering the potential importance of viruses in natural environments, understanding what role viruses play in the C, N, and P cycles and the impact of pathogenic genes in the genome of viruses on public safety is important in campus wastewater treatment plants. This study used metagenomic technology to analyse in depth the human bacterial and viral pathogens that may be present in sludge, along with the ARGs, virulence factors, and toxins located on viruses, and analysed the potential functions of bacteria and viruses in the element cycle. Our study demonstrates that a large number of pathogenic bacteria and unknown viruses occur in campus wastewater treatment plants (WWTPs), and the ARGs, virulence factors, and toxins in the genome of viruses pose potential health risks. The auxiliary metabolic genes involved in the C, N, and P cycles located on viruses in the sludge are beneficial to the cycling of elements in the sludge. In contrast to previous studies that primarily concentrated on bacteria and archaea, this research endeavors to redirect attention towards elucidating the potential ecological functions, and discerning the concomitant public health risks posed by viruses and bacteria within wastewater treatment plants.

## 2. Materials and Methods

### 2.1. Sample Collection

The activated sludge samples were collected on 22 October 2021 from two wastewater treatment plants located 360 m apart at a university in China. These plants primarily receive domestic wastewater from dormitories and canteens. A total of six 10 L activated sludge samples (WTP1-1, WTP1-2, and WTP1-3 from Plant 1; WTP2-1, WTP2-2, and WTP2-3 from Plant 2) were obtained using acid-washed polycarbonate bottles. Subsequently, the samples were promptly transported to the laboratory within a time frame of four hours at room temperature.

### 2.2. DNA Extraction

The sludge sample was initially filtered through an 800-mesh nylon filter followed by a 0.22 μm filter membrane (Millipore, Burlington, MA, USA) to separate viruses from prokaryotes. Subsequently, the membranes containing prokaryotic cells were harvested, frozen with liquid nitrogen, pulverized, and subjected to extraction of total prokaryotic DNA using the DNeasy Power Soil Pro Kit (QIAGEN, Hilden, Germany). The virus-containing filtrate was mixed with PEG 8000 and NaCl to achieve final concentrations of 10% (*w*/*v*) and 0.5 M respectively before being stored at 4 °C overnight. After centrifugation at 10,000× *g* for 30 min, the supernatant was discarded and the pellet was resuspended using SM buffer solution. DNase I (final concentration of 10 U/mL, TaKaRa, Beijing, China) and RNase A (final concentration of 200 μg/mL, TaKaRa, China) were added, and incubated at 37 °C for 2 h. After treatment at 80 °C for 5 min, the mixture was supplemented with proteinase K, EDTA, and SDS, and then incubated at 55 °C for 3 h. Viral DNA was extracted using a phenol-chloroform-isoamyl alcohol method as described by Che in 2022 [11].

### 2.3. 16S rRNA Gene Amplicon and Virome Sequencing

The prokaryotic 16S rRNA gene was amplified using 341F/806R primers and sequenced on the Illumina Miseq PE300 platform at Shanghai Majorbio Bio-pharm Technology Co., Ltd. The dada2 plugin in Qiime2 was utilized for denoising paired-end sequencing data of 16S rRNA gene sequences, resulting in the generation of ASV (Amplicon Sequence Variant) tables and feature sequences. The q2-feature-classifier plugin and Silva 16S rRNA database (v138) [12] were employed to train a Naive Bayes classifier specifically for V3–V4 sequences, enabling the classification of prokaryotic species. Chloroplast and mitochondrial sequences were eliminated, along with ASVs having a total count < 10. Multiple bacterial pathogen detection pipeline (MBPD) [13] was applied to detect zoonotic pathogens in animals, plants, and humans. It employs the mothur software (v1.45.3) for sequence alignment, with an identity threshold established at 0.8. Viral DNA underwent sequencing on the Illumina PE150 platform. Briefly, viral DNA was randomly fragmented using an ultrasonic homogenizer and used for constructing the sequencing library; subsequent sequencing occurred after passing quality control measures. Trimmomatic (v0.32) [14] was utilized to eliminate paired-end reads containing adapters. Corresponding paired reads were also discarded if the number of low-quality bases (SQ ≤ 20) exceeded 20% of the total read bases in single-ended sequencing data. Duplicate reads generated by PCR amplification as well as polyX sequences were removed.

### 2.4. Identification of Viral Contigs

The clean reads were assembled into contigs using the metaSPAdes software (v3.15.4) [15]. Subsequently, contigs with a length greater than 2500 bp were selected for further analysis. The contigs underwent initial filtering and screening using VirSorter2 [16] with a threshold of 0.5 to select ssDNA and dsDNA viral sequences. False-positive viral sequences were subsequently eliminated using CheckV [17]. The potential viral sequences were then de-replicated utilizing CD-HIT-est [18] with a threshold of 90% coverage and 95% confidence, resulting in non-redundant sequence sets for each sample.

### 2.5. Annotation of Virome and Host Prediction

The clean reads underwent annotation for viral species employing the Kraken2 software and the corresponding viral database [19] based on the k-mer algorithm. The annotation results were filtered through Bracken [20] and visualized via Pavian [21]. Human viruses were identified by screening against the Virus–Host DB [22]. Phage lifestyles were determined using PhaTYP [23]. Contigs were classified and annotated utilizing PhaGCN [24], while host prediction for viral sequences was performed using CHERRY [25].

### 2.6. Viral Gene Functional Prediction

The Prokka software [26] was utilized to predict genes in the obtained non-redundant viral sequence set based on viral models. Subsequently, CG-HIT was employed with 90% coverage and 95% identity as thresholds to eliminate redundant predicted protein sequences, resulting in a non-redundant protein sequence set. Carbohydrate active enzymes were annotated using the run_dbcan script of dbCAN2 [27]. To annotate nitrogen metabolism genes and phosphorus metabolism genes, the protein sequence sets were compared to the NCycDB [28] database and PCycDB [29] database, respectively, employing Diamond [30] with default parameters. PathoFact [31] was applied for predicting pathogenic factors such as ARGs, toxin genes, and virulence factors. Furthermore, virulence factors were annotated by comparing the dataset to VFDB’s core dataset using Diamond. [32].

## 3. Results

### 3.1. Pathogenic Bacteria within WWTP

**Figure 1:**
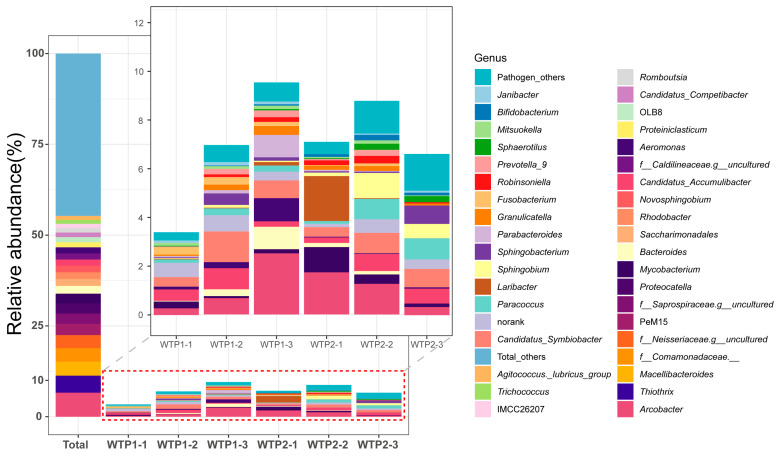
The relative abundance of bacterial genera in all samples and the bacterial pathogen in each sample.

A total of 654,766 original 16S rRNA gene reads were obtained from six samples collected from two WWTPs; these reads included 25 phyla, 49 classes, 113 orders, 188 families, 308 genera, and 1334 ASVs (Figure 1).

MBPD was used to analyse the pathogenic bacteria in the samples (Figure 1). A total of 242 ASVs were identified as pathogenic bacteria, with animal pathogens accounting for 41.22% of the total number of sequences. All the pathogenic bacteria were distributed in 13 phyla, of which Proteobacteria, Campylobacterota, Bacteroidota, Firmicutes, Patescibacteria, and Actinobacteriota were the dominant phyla, accounting for 18.61%, 6.79%, 5.41%, 3.64%, 3.21%, and 3.20% of the total number of sequences, respectively.

The pathogenic Proteobacteria included *Candidatus symbiobacter* (4.36%), *Candidatus accumulibacter* (3.11%), *Paracoccus* (2.50%), *Laribacter* (2.07%), *Sphingobium* (2.00%), and *Aeromonas* (1.46%). The pathogenic bacteria in the phylum Campylobacterota were all *Arcobacter* and had the highest relative abundance (6.79%). The phylum Bacteroidota included the genera *Bacteroides* (1.51%), *Sphingobacterium* (1.50%), and *Parabacteroides* (1.20%). The phylum Firmicutes included genera such as *Granulicatella* (1.05%). Pathogenic cases of *Granulicatella* species are relatively rare, but they can cause diseases such as bacteremia, sepsis, and infective endocarditis [33]. *Mycobacterium* (2.08%) was the dominant pathogen in the phylum Actinobacteria.

In addition to bacteria, a small number of archaea were also identified in the samples, accounting for 0.18% of the total number of sequences. They were all classified into three genera, *Methanosaeta*, *Methanobrevibacter*, and *Methanomethylovorans*, under Methanosarcinaceae.

### 3.2. Viral Community Composition within WWTP

After metavirome sequencing and quality control, 270,227,598 reads were obtained. After Kraken annotation, a total of 455,846 reads were annotated as viral sequences; these reads belonged to 8 phyla, 12 classes, 20 orders, 63 families, and 613 genera.

**Figure 2:**
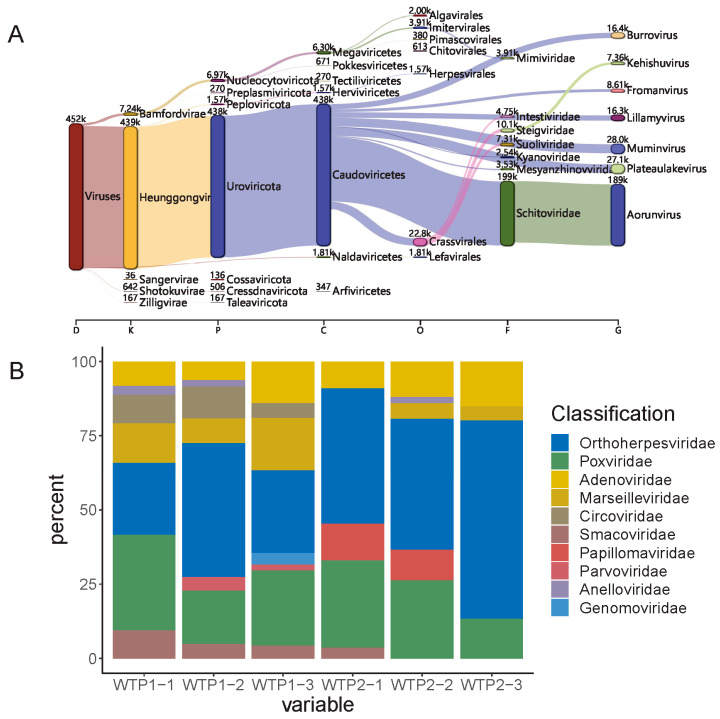
Viral composition and the relative abundance of pathogenic viruses. (**A**) Viral composition of all samples. Number means the number of reads classified in each level. (**B**) Relative abundance of the dominant pathogenic viral families among all viral sequences.

*Aorunvirus* (41.85%), *Muminvirus* (6.20%), *Plateaulakevirus* (6.00%), *Burrovirus* (3.63%), *Lillamyvirus* (3.61%), *Fromanvirus* (1.91%), *Kehishuvirus* (1.63%), and *Nipunavirus* (1.14%) were the dominant viral genera in the samples (Figure 2A), and all belonged to the class Caudoviricetes. There were considerable differences in the compositions of the viral communities among the two WWTPs, as shown by the fact that *Aorunvirus* accounted for only 0.19%, 0.23%, and 0.07% of the number of sequences in the WTP1-1, WTP1-2, and WTP1-3 samples but 49.65%, 73.63%, and 39.65% of the total number of sequences in the WTP2-1, WTP2-2, and WTP2-3 samples, respectively. However, the distribution pattern of *Plateaulakevirus* was opposite that of *Aorunvirus*; that is, it accounted for a relatively high proportion of the total number of sequences in the WTP1-1, WTP1-2, and WTP1-3 samples, i.e., 28.99%, 7.78%, and 17.13%, respectively, but a relatively small proportion in the other three samples, accounting for 0.14% of the total number of their respective sample sequences. All the other viral genera were found with similar content and without significant differences among the six samples.

The annotation results were screened using the Virus-Host Database (V-H DB). A total of ten viral families that can infect humans, including Orthoherpesviridae, Poxviridae, and Adenoviridae, were identified, accounting for 0.66% of the total number of viruses. Among them, Orthoherpesviridae accounted for 0.25% of the total number of species (Figure 2B).

### 3.3. Viral Lifestyle and Host Prediction

Phage lifestyle prediction was performed using PhaTYP. There were 25,770 contigs with scores higher than 0.9, in which virulent phages accounted for 60.94% of the total number of contigs and temperate phages accounted for 39.06%, indicating that virulent phages are dominant in sludge.

**Figure 3:**
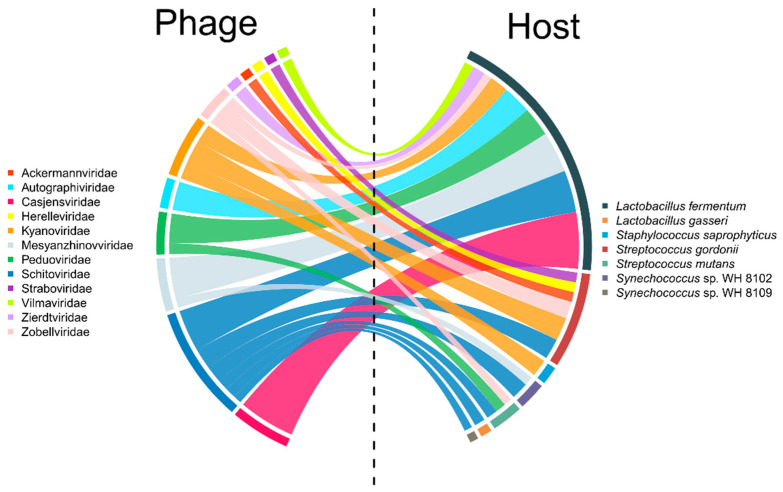
Predicted hosts of viral contigs from WWTPs.

Using PhaGCN and CHERRY to predict viral hosts, we found that in the samples, the phage hosts were mainly *Lactobacillus*, *Streptococcus* and *Staphylococcus*, accounting for 37.11%, 21.11%, and 7.11%, respectively (Figure 3).

*Lactobacillus fermentum* was the host for 18 phage species in the samples, among which Casjensviridae, Schitoviridae, and Mesyanzhinovviridae were the most abundant, accounting for 21.14%, 15.77%, and 15.44%, respectively. Notably, the abundances of Schitoviridae and Mesyanzhinovviridae viruses were also high (ranking first and sixth in abundance, respectively) according to the viral species annotation results generated via Kraken for metavirome sequencing.

Using an integrity greater than 95% as the threshold, a total of 241 complete viruses were obtained from the six samples, accounting for 0.51% of the total number of contigs. Among them, 72 sequences were longer than 50 kb in length, accounting for 29.99% of the complete viruses. Annotation and host prediction were performed for 72 complete viruses, and 23 sequences had a PhaGCN annotation score greater than 0.9. These 23 sequences belonged to six families, including Schitoviridae, Mesyanzhinovviridae, and Zobellviridae, and included bacteria from eight genera, including *Synechococcus*, *Streptococcus*, and *Lactobacillus*, as hosts. The annotation scores of the remaining 49 sequences were low; therefore, they may be new viruses that have not yet been discovered. The two sequences with the lowest scores in species annotation were selected and compared to the NCBI database using BLASTn. The results showed that the query cover values were both between 0% and 4%, suggesting that there are abundant novel viruses in the sludge.

### 3.4. Viral Genes Related to Carbon, Nitrogen, and Phosphorus Metabolism

**Figure 4:**
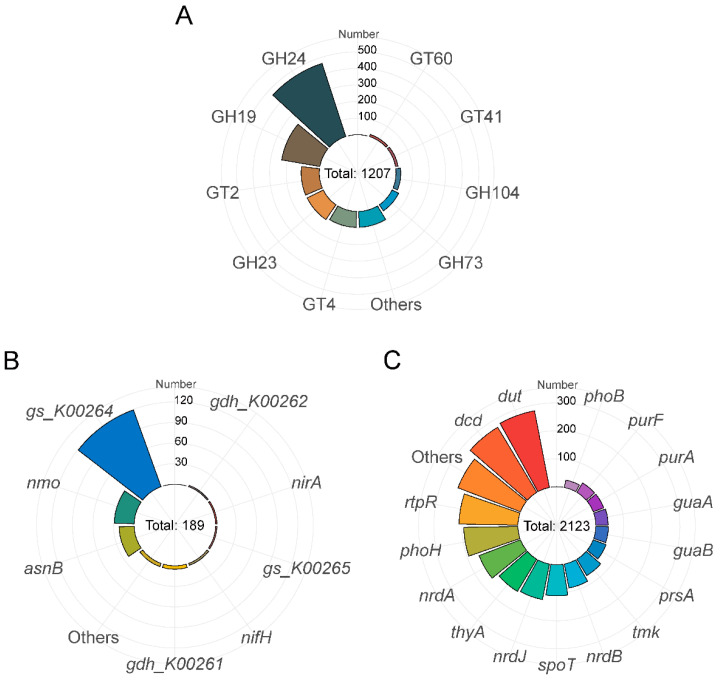
The number of viral AMGs that participate in the carbon (**A**), nitrogen (**B**), and phosphorus (**C**) cycles.

Five types of carbon metabolism genes, i.e., carbohydrate-binding module family, carbohydrate esterase family, glycoside hydrolase family, glycosyltransferase family, and polysaccharide lyase family genes, were detected, for a total of 34 types of genes related to the metabolism of 1207 carbohydrate-active enzymes (Figure 4A). The glycoside hydrolase family and the glycosyltransferase family were most abundant, with 900 and 295 genes, respectively.

The prediction results for genes involved in nitrogen metabolism showed that the viruses contained 379 nitrogen metabolism genes in 13 species (Figure 4B), including *gs_K00264*, *nmo*, *asnB*, *gdh_K00261*, *nifH*, *gdh_K00262*, *nirA*, and *gs_K00265*. Among these genes, *gs_K00264* accounted for the highest proportion (62.96%) of the total number of genes involved in nitrogen metabolism. *gs_K00264* is a component of the organic degradation and synthesis pathway and encodes glutamate synthase (NADPH/NADH), which is a complex iron-sulfur protein involved in the assimilation of ammonia [34].

A total of 2123 genes of 60 types were obtained from the prediction of viral genes involved in phosphorus metabolism (Figure 4C) and were involved in metabolic pathways such as pyrimidine metabolism, purine metabolism, the pentose phosphate pathway, and oxidative phosphorylation, with *dut*, *dcd* and *rtpR* being the three most abundant genes. In addition, these genes included those encoding adenylate kinase (*adk*) and guanylate kinase (*gmk*), which are involved in purine metabolism. Moreover, phosphatases such as phosphatase R (*phoR*) and phosphatase B (*phoB*) are also predicted to participate in the removal of phosphate.

### 3.5. Antibiotic Resistance Genes, Virulence Factors, and Toxin Genes Located on Viruses

Three pathogenic factors: antibiotic-resistance genes, virulence factor-encoding genes, and toxin genes, were analysed using PathoFact. A total of 217 ARGs (113 types) were detected, 942 virulence factor-encoding genes (180 types) were detected, and 788 toxin genes (32 types) were detected.

**Figure 5:**
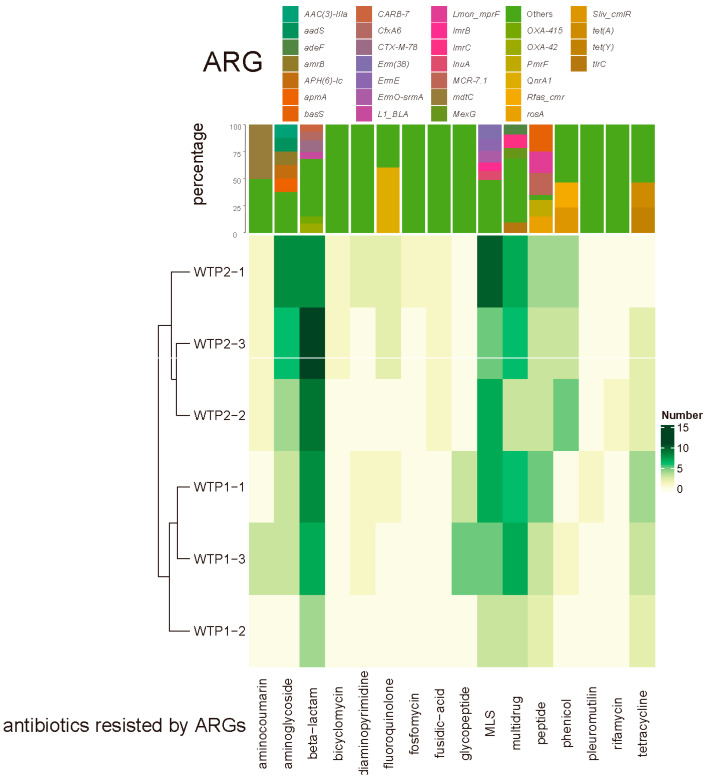
Composition (above) and number (below) of detected ARGs in the virome within WWTPs.

Among the 113 detected ARGs, 16 major classes and 39 subclasses of antibiotics developed resistance, helping the carrier bacteria resist antibiotics mainly through mechanisms such as antibiotic efflux or inactivation (Figure 5). ARGs resistant to beta-lactam and macrolide, lincosamide, and streptogramin (MLS) antibiotics had the highest relative abundances, accounting for 21.6% and 17.1%, respectively, of the total number of ARGs. The predicted 217 ARGs were distributed on 216 viral contigs. These viruses infect 21 genera of bacteria, mainly *Lactobacillus* (34.88%), *Streptococcus* (12.80%), and *Staphylococcus* (11.63%), which is consistent with the distribution pattern of previous host prediction results for all the samples. These results suggest that these bacteria may acquire and transmit ARGs through horizontal gene transfer, posing hidden dangers to public health security.

**Figure 6:**
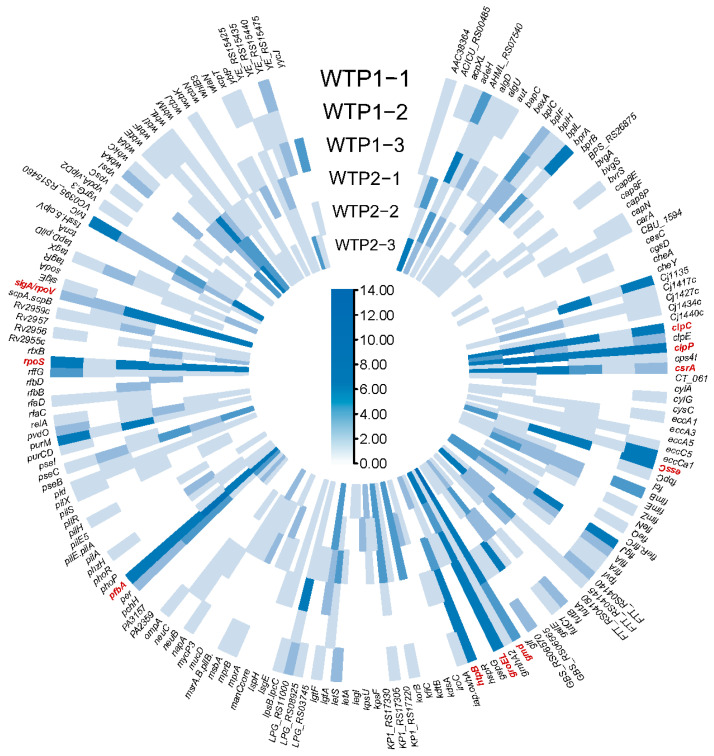
Heatmap representing the abundance of viral genes assigned to the virulence factor count of each sample. The red font indicates dominant genes.

The virome contained 942 genes encoding 180 virulence factors (Figure 6). Regardless of the genes encoding potential virulence factors or the determined virulence factors, the number of non-secretable virulence factor-encoding genes was relatively large, with a total of 909 genes. A total of 96.50% of the genes were predicted to be virulence factors. These included *clpP* (4.78%), *pfbA* (4.67%), *gmd* (3.72%), *clpC* (3.72%), *rpoS* (3.40%), *sigA/rpoV* (3.40%), *groEL* (3.18%), *csrA* (2.44%), *essC* (2.12%), and *htpB* (2.02%). *clpP* encodes the ATP-dependent Clp protease proteolytic subunit, a proteolytic enzyme that helps pathogens escape from the phagocytosis of macrophages in the host, thereby improving the survival ability of pathogens [35]. The *pfbA* gene encodes a surface adhesin that binds to human fibronectin and plasminogen in the host extracellular matrix. Its function is mainly related to the adhesion and invasion of the host by pathogenic bacteria, which are mediated by adhesin and invasion [36].

**Figure 7:**
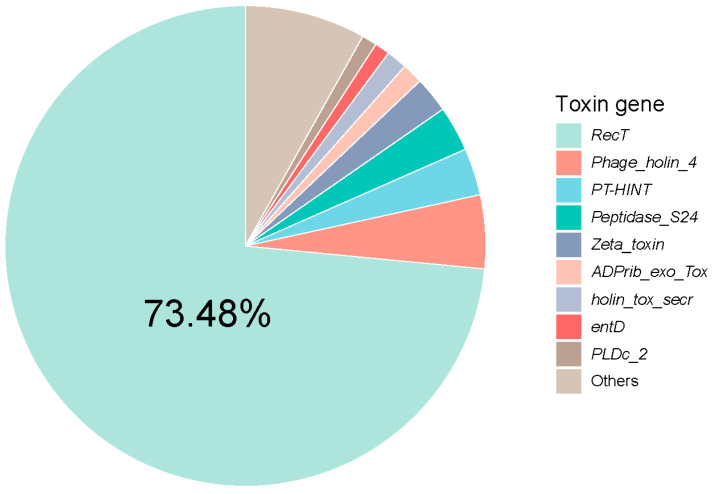
Relative abundance of viral genes assigned to toxin within all samples.

The virome contained a total of 32 toxin genes, including *RecT* (73.48%), *Phage_holin_4* (4.95%), *PT-HINT* (3.17%), *Peptidase_S24* (3.05%), *Zeta_toxin* (2.41%), *ADPrib_exo_Tox* (1.40%), *holin_tox_secr* (1.40%), *PLDc_2* (1.02%), and *entD* (1.02%) (Figure 7). *RecT* is a DNA single-strand annealing protein (SSAPs) that belongs to the RecT family and plays a role in the process of DNA recombination [37].

## 4. Discussion

Previous studies on the function of microorganisms in wastewater treatment systems have focused mainly on bacteria and archaea. Viruses are the most abundant entities in sludge, yet very little is known about the assemblage composition, both in terms of taxonomy as well as genetic makeup. This study used metagenomic technology to elucidate the potential ecological functions and public health risks of bacteria and viruses in the WWTPs.

This study has shown that bacteria such as Proteobacteria, Campylobacterota, Bacteroidota and Firmicutes are relatively highly abundant in WWTP. These findings are consistent with several previous result [38,39,40]. In this study, up to 41% of the bacterial sequences were potential animal pathogens, with the most abundant pathogen being the genus *Arcobacter* in the phylum Campylobacterota, accounting for 6.79% of the total number of sequences. This strain has been reported in poultry, livestock, pork and other meat products, and vegetables and can cause enteritis, severe diarrhoea, septicemia, and miscarriage in humans [41,42,43]. Moreover, it has been reported that this bacterium is significantly associated with various ARGs, such as *tetA*, *sul1*, and *int1*, and is a potential multidrug-resistant pathogenic bacterium [39]. The results of viral host prediction revealed that the hosts with the most viral infections were all Firmicutes, not Proteobacteria, which accounted for the greatest proportion. It is speculated that the phages of the Caudoviricetes class were generally the most abundant.

Compared with that of bacteria, the composition of the viral communities in WWTPs in different areas varies greatly, but as the results of this study show, phages of the Caudoviricetes class generally occupy a dominant position. We found that the sludge contained 0.66% human pathogenic viruses, with members of the families *Orthoherpesviridae* and *Poxviridae* being the two most common pathogenic viruses. This result was consistent with the findings of other studies on lakes or wastewater [11,44]. *Orthoherpesviridae* viruses are double-stranded DNA viruses that often infecting amniotes. Infection can cause various diseases, such as shingles, chickenpox, and genital herpes [45]. Viruses of the family *Poxviridae* are also a type of double-stranded DNA virus that can cause purulent lesions on the skin throughout the body after infecting humans [46]. Although existing research has shown that the proportion of pathogenic viruses in sludge is less than 1% [47], it is generally believed that viruses have a greater impact on public health and safety compared with pathogenic bacteria and protozoa in wastewater [48,49,50]. Additionally, safety concerns about the application of reclaimed water have been raised [51,52].

The ARGs located on phages can be transmitted between hosts through horizontal gene transfer, posing a risk to public health [53]. We detected various ARGs belonging to the OXA family, including *OXA-42*, *OXA-46*, and *OXA-119*, most of which are plasmid-mediated β-lactamases that can hydrolyse related antistaphylococcal penicillins, oxacillin, and other antibiotics. Due to possible contamination by faeces or hospital wastewater, β-Lactam and MLS antibiotics are also ubiquitously found in wastewater treatment systems worldwide [54]. Virus-mediated horizontal transfer of ARGs can enhance the viability of microbial communities in wastewater treatment systems, affecting the treatment effect of wastewater treatment systems on pathogenic bacteria. Moreover, various virulence factors and toxins encoded and expressed by viruses are involved in the host infection process. The lysozyme encoded by *clpP* can help the pathogen evade the host immune response, and DNA single-strand binding proteins of the *RecT* family promote viral replication. The holin proteins produced by phages are involved in host cell lysis. Phages accumulate the holin protein at a certain concentration, causing rupture of the host cell membrane and the release of endolysin, which hydrolyses the cell wall, thus leading to bacterial infection [55]. The presence of these factors enhances the ability of viruses to infect bacteria and promotes gene flow between microbial communities. This process facilitates the spread of functional genes required for pollutant degradation and nutrient transformation among microbial communities but may also accelerate the spread of related disease-causing genes.

Viruses are involved in the metabolism of key nutrients, such as carbon, nitrogen, and phosphorus in wastewater. Viral carbon-metabolizing enzymes, such as glycoside hydrolases and glycosyltransferases, as well as nitrogen- and phosphorus-metabolizing enzymes, such as glutamate synthase and dUTPase, are involved in processes such as the degradation of sugars and nucleotide metabolism and are closely related to viral replication and transmission. The glycoside hydrolase located on viruses helps the host decompose complex carbohydrates and generate energy, it promotes the growth and reproduction of the host, and improves the phage’s living conditions [56]; moreover, this hydrolase is conducive to the migration of organic matter in wastewater [57]. We found that the most abundant gene involved in nitrogen metabolism located on viruses was glutamate synthase, which is related to biofilm formation in bacteria [58]. In sludge, phage infection may enhance the ability of the host to form biofilms.

Phages also carry numerous phosphorus metabolism genes involved in the synthesis of progeny viruses. Nucleic acid synthesis is an important step in the production of progeny viruses. Within the viral genomes, we detected many genes involved in purine and pyrimidine metabolism. *rtpR* can reduce ribonucleoside triphosphate to deoxyribonucleoside triphosphate, which, after being catalysed by *dcd*, can deaminate dCTP to form dUTP. After catalysis by *dut*, dUTP removes a molecule of phosphoric acid to form dUMP. In addition, *thyA* and *tmk* are involved in this phosphorus metabolic pathway. The enzymes encoded by several genes involved in phosphorus metabolism form a pyrimidine synthesis pathway from CTP to dTDP, mediate the production of various nucleotides, and facilitate the transmission of viral genetic information and the generation of progeny viruses. In addition, several phosphate degradation- and biological respiration-related enzymes in this network play a role in the degradation of organic matter in wastewater. The presence of these genes is important for the stable and efficient degradation of substances in WWTPs. In addition to encoding auxiliary metabolic genes, viruses also affect wastewater treatment by lysing microorganisms involved in nutrient transport and element cycling [57]. In conclusion, viruses significantly regulate the function of microorganisms in sludge by transporting a variety of auxiliary metabolic genes and lysing their hosts.

## 5. Conclusions

We used metagenomic technologies to analyse the community composition and potential functions of bacteria and viruses in two WWTPs. The results revealed an abundance of pathogenic bacteria and pathogenic viruses in the sludge. The diverse pathogenic genes, such as antibiotic-resistance genes, virulence factors, and toxin genes, contained in the viruses have nonnegligible health risks for water reuse. Additionally, the diverse auxiliary metabolic genes contained in the viruses can regulate the metabolism of C, N, and P in the hosts, indirectly affecting wastewater treatment efficacy. Bacteriophages in sludge are not only carriers of harmful genes, but also an important resource bank, from which bacteriophages infected with pathogens can be isolated. One of the key challenges for future research on sewage is how to effectively reduce safety hazards and increase the utilization efficiency of reclaimed water and its microbial resources.

## Data Availability

Data is deposited in National Microbiology Data Center (NMDC) with accession numbers NMDC40053725-NMDC40053736.
